# Understanding immune perspectives and options for the use of checkpoint immunotherapy in HCC post liver transplant

**DOI:** 10.20517/2394-5079.2021.123

**Published:** 2022-02-11

**Authors:** Chimaobi M. Anugwom, Thomas M. Leventhal, Jose D. Debes

**Affiliations:** 1 HealthPartners Digestive Care, Saint Paul, Minnesota, MN 55130, USA.; 2 Department of Medicine, Division of Gastroenterology, Hepatology and Nutrition and Division of Infectious Disease and International Medicine, University of Minnesota, Minneapolis, MN 55455, USA.; 3 Department of Gastroenterology and Hepatology, Erasmus Medical Center, Postbus 2040, The Netherlands.

**Keywords:** Hepatocellular carcinoma, liver transplant, checkpoint inhibitors

## Abstract

Treatment modalities for hepatocellular carcinoma (HCC) vary from surgical techniques and interventional radiologic strategies to systemic therapy. For the latter, the use of immune checkpoint inhibitors (ICIs) has gained popularity due to successful trials showing increased survival. In patients who have undergone liver transplantation, recurrence of HCC poses a significant challenge. There is indeed considerable debate on the efficacy and safety of ICI use in liver transplant recipients due to competing immune interests in maintaining a healthy graft and combating the tumor. Recent reports and case series have highlighted a role for the type of immune therapy, timing of therapy, tissue expression of PD-1 and modulation of immunosuppression, in the understanding of the efficacy and risks of ICIs for HCC in liver transplant. In this article, we appraise the available literature on the usage of ICIs for HCC in liver transplant recipients and provide perspectives on immune concerns as well as potential recommendations to consider during the management of such complex cases.

## INTRODUCTION

Hepatocellular carcinoma (HCC) is the predominant primary liver malignancy - representing about 75% of all primary liver cancers^[1]^. Consequently, HCC causes a significant global public health care burden, as it is the seventh most common malignancy and the second most common cause of cancer-related mortality worldwide^[2]^.

In the majority of individuals, HCC occurs as a complication of underlying chronic liver disease. Globally, hepatitis B is the most important risk factor for developing HCC, while hepatitis C, alcohol-related liver injury and non-alcoholic fatty liver disease represent prominent etiological risk factors in resource-rich countries^[3–5]^.

Treatment modalities for HCC include surgical resection, ablation therapies including radiofrequency ablation, microwave ablation and electroporation; as well as, in select candidates, liver transplantation (LT)^[6–9]^. Resection or ablation of HCC, in those deemed appropriate candidates, can lead to long-term disease-free survival and LT can provide the additional benefit of not only removing the malignancy but eliminating underlying chronic liver disease as well. In patients with HCC, despite the use of strict selection criteria for candidates for LT, there remains a risk of HCC recurrence in the transplant recipient^[6,10]^. The mean rate of HCC recurrence after LT is about 16%, and can be as high as 20%, with 75% of cases occurring within the first two years of the post-transplant period^[10–12]^. Even more concerning is the dramatic course of tumor recurrence. It is considered a systemic event, as the transplanted liver alone is involved in only 30% of cases while approximately 50% of cases of HCC recurrence post-LT involves multiple organs: the lungs, skeletal system, and adrenal glands being the most common sites of recurrence^[13,14]^.

**Figure 1.**
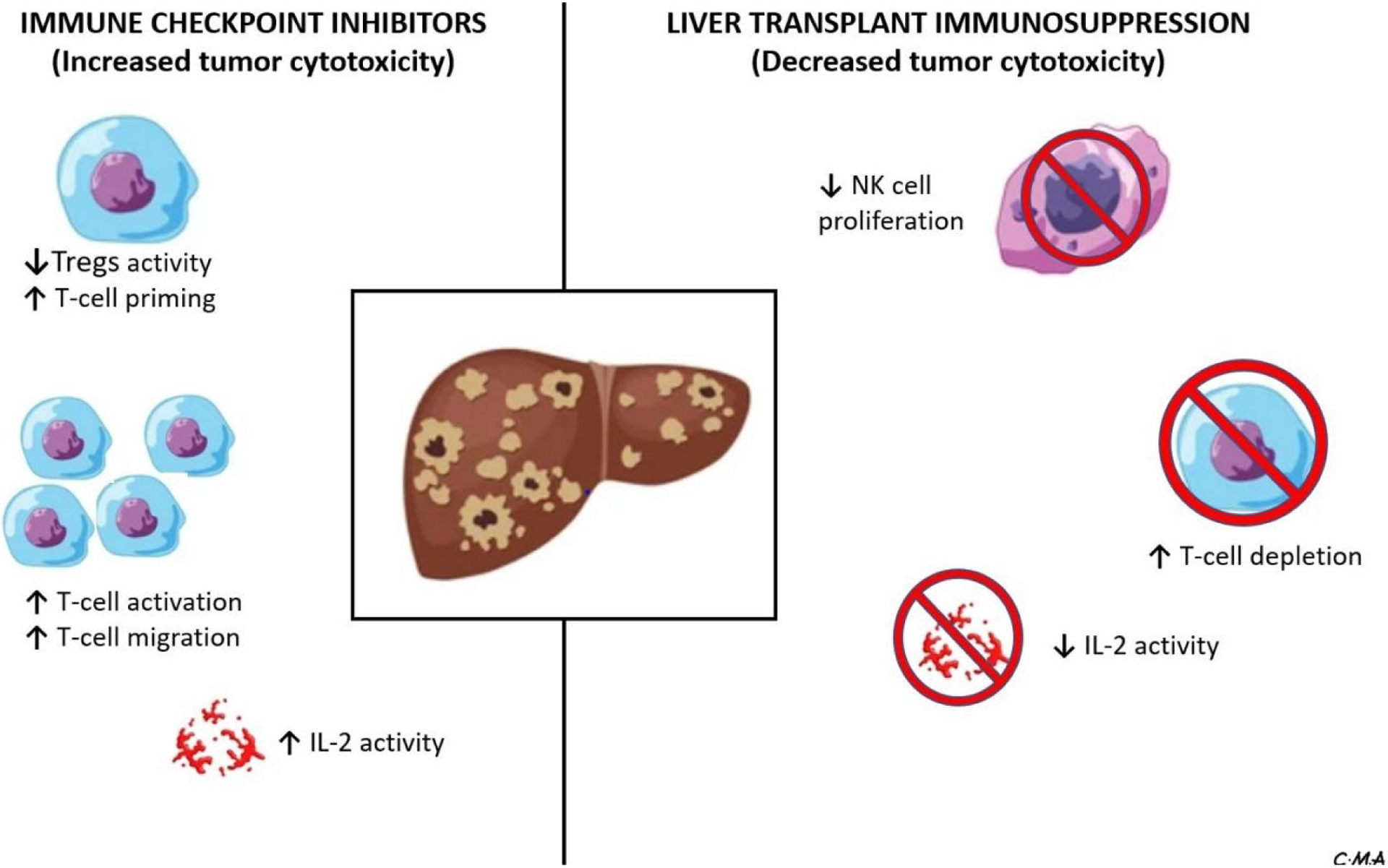
Interplay between immune checkpoint inhibitors and liver transplant immunosuppression in a recipient with hepatocellular carcinoma, emphasizing the effect of these medications on components of the immune system. NK: Natural killer; IL: interleukin.

The great strides in cancer therapy in recent years include the emergence of immunotherapeutic agents, which have become commonplace in the management of most cancers, including HCC^[15]^. Indeed, over the last few years, checkpoint immunotherapy for HCC has advanced at an explosive pace and despite the cost of treatment (including copays, office visits, and laboratory tests), the cost of management of adverse events and its contribution to the overall cost of cancer care; it is now considered first-line therapy for advanced HCC in those individuals that can tolerate it^[16]^. There is, however, still ongoing debate about the safety and efficacy of these medications in patients who have undergone LT, given the contrasting mechanism of action of these immunotherapeutic agents compared to immunosuppression for LT [Figure 1]. Moreover, there is an incomplete understanding of the effects caused by the inter-relation between the non-cancer-related activation of immune exhaustion triggered by immune-therapy and immune-modulation related to anti-rejection medications in these patients. In this review, we discuss critical aspects of checkpoint immunotherapy for HCC following LT based on existing data and as well as providing insight into controversial issues in the field.

## CHECKPOINT IMMUNOTHERAPY IN THE TREATMENT OF HEPATOCELLULAR CARCINOMA

Many drug classes are employed in the systemic treatment of hepatocellular carcinoma. Sorafenib was the first agent to demonstrate survival benefit as a first-line therapy for unresectable HCC based on the SHARP and Asian-Pacific trials and remained the sole resource for advanced HCC for over ten years^[17,18]^. Sorafenib is a multi-kinase inhibitor that acts by inhibiting a variety of tyrosine-kinase receptors, including vascular endothelial growth factor receptor and platelet-derived growth factor receptor and has mainly been shown to be effective in selected patients such as those with hepatitis C and favorable neutrocyte-lymphocyte ratio^[19–21]^. Additional therapies such as lenvatinib, regorafenib, and cabozantinib, all targeting a combination of tyrosine kinase receptors, as well as ramicirumab with specific targeting of VEGF, have been approved for the treatment of HCC^[22–25]^.

Although lymphocyte infiltration of HCC is variable, its presence, as reported by Yoong *et al*.^[26]^, may allude to the apparent immunogenicity of this tumor, thus making immunotherapy an exciting prospect for HCC therapy. This can be accomplished by either removing barriers to the body’s natural immune response to tumor antigens - in the case of checkpoint inhibitors - or by stimulating a novel response by targeting specific HCC antigenic molecules.

During T-lymphocyte activation, binding of the programmed death ligand-1 (PD-L1) to the PD-1 receptor or the cytotoxic T-lymphocyte associated antigen-4 (CTLA-4) to B7-1/B7-2, produces a co-inhibitory signal, which prevents lymphocytes from attacking specific host cells^[27,28]^. Tumor cells may hijack these checkpoint mechanisms, thus escaping immunologic surveillance. Immune checkpoint inhibitors (ICIs), as the name indicates, are immunotherapeutic agents that are effective in targeting and inhibiting these checkpoints, thereby activating T-lymphocytes, and potentially those with anti-tumoral activity^[28]^. By removing this co-inhibitory signal, ICIs augment the immune response toward the tumor. However, removal of these checkpoints, in addition to the subsequent activation of other non-specific T-cells, lead to an increased risk of immune-related adverse effects (IRAEs) in the host^[29]^. The various ICIs approved or being studied for the systemic treatment of HCC are shown in Table 1.

Single-agent treatment of HCC with the PD-L1/PD-1 inhibitors comprises most of the initial data on this topic. Nivolumab and pembrolizumab (both PD-1 inhibitors) have shown significant promise in the treatment of HCC and were initially approved for use as second-line therapy in patients who have been exposed to sorafenib. The CheckMate-040 study was a single-arm, non-comparative, dose escalation and expansion trial showing median overall survival of 7.6 months with Nivolumab in patients exposed to Sorafenib. This led to the approval of nivolumab for the indication of treatment of HCC by the FDA in 2017^[30]^. In the KEYNOTE-240 trial, pembrolizumab showed a median survival of 13.9 months compared to 10.6 months with placebo [Hazard ratio (HR) = 0.78]^[31]^. Nivolumab was studied as a first-line agent compared to sorafenib in the CheckMate-459 study. Although there was some evidence of increased survival in the Nivolumab group, this study did not meet its primary endpoint of statistically significant improvement in overall survival^[32]^.

The combination therapy with atezolizumab (PD-L1 inhibitor) and bevacizumab (Anti-VEGF) was compared to sorafenib in the IMbrave study^[33]^. This was a multicenter, randomized, phase III open-label trial, that showed a median progression-free survival of 6.8 months with atezolizumab/bevacizumab compared to 4.3 months with sorafenib (HR = 0.59), as well as increased overall survival (HR = 0.58) in the atezolizumab/bevacizumab group^[33]^. This led to the approval of this combination by the FDA as first-line therapy for advanced HCC in 2020. Sintilimab (PD-L1 inhibitor) combined with a bevacizumab biosimilar (a biologic medical product highly similar to the already approved biological) has been compared to sorafenib in the ORIENT-32 trial. The overall survival and progression-free survival were both higher in the sintilimab/bevacizumab-biosimilar group (HR = 0.57 for both outcomes)^[34]^. Other combinations including ICIs in ongoing trials include: atezolizumab/cabozantinib (COSMIC-312, NCT03755791), lenvatinib/pembrolizumab (LEAP-002, NCT03713593) nivolumab/ipilimumab (CHECKMATE-9DW, NCT04039607) and durvalumab/bevacizumab (EMERALD-2, NCT03847428)^[35]^. It is expected that these additional ICIs will expand the immunologic treatment options for patients with HCC in the near future.

## THE IMMUNOLOGICAL MILLEU OF THE TRANSPLANT RECIPIENT

Immunosuppression is essential to long-term patient and graft survival after LT. Compared to transplantation for other solid organs, the liver is quite immune tolerant, and this is related to the unique immunologic microenvironment created by the liver-derived dendritic cells, liver sinusoidal endothelial cells, liver-derived natural killer cells, and Kupffer cells^[36]^. This unique environment is crucial in maintaining organ homeostasis and keeping a balance between immune tolerance and inflammation when exposed to infectious and tumorigenic triggers^[37,38]^. In the LT recipient, this unique immune-environment may explain the need for less overall systemic immunosuppression and potential for immunosuppression withdrawal after LT^[38,39]^.

There has been significant advancement in the strategies aimed at successfully preventing rejection of the allograft since the first successful liver transplantation by Starzl *et al*.^[40,41]^ in the 1960’s. In the early days of LTs, corticosteroids and azathioprine were used as the primary immunosuppression strategy and this has evolved to more recent immunosuppression modalities such as calcineurin inhibitors (CNIs), anti-metabolites, mammalian targets of Rapamycin inhibitors (mTORs), T-cell depleting and T-cell inhibiting antibodies^[42,43]^.

The consequent effect of transplantation on the native immune system is the reduction of T-cell stimulation, proliferation and differentiation, impairment of natural killer cell proliferation, and significant downregulated production of co-stimulatory molecules by antigen-presenting cells with a decrease in the production of pro-inflammatory cytokines^[44–46]^. These changes, though necessary for long-term allograft survival, have a deleterious effect on the ability of the immune system to actively detect and attack cancer cells, so it is no surprise that the risk of some malignancies increases in the post-LT period. As previously alluded to, in those transplanted for HCC, tumor recurrence can be as high as 20%, and this risk is affected by immunosuppression, obesity, donor age, etiology of liver disease^[47–49]^. The *de-novo* cancer risk in patients post-LT, based on over 108,000 recipients between 1987 and 2015, was obtained from the United States Scientific Registry of Transplant Recipients database, and this estimated the cumulative incidence of *de novo* extrahepatic cancer to be about 1.3% (95%CI: 1.3–1.4) in the first year after LT; and up to 18.8% (18.4–19.3) at 20 years^[47]^. The most common *de-novo* malignancies in the LT population include Non-Hodgkin’s lymphoma, keratinocyte skin cancer (basal cell cancer and squamous cell cancer), cervical cancer and head/neck cancers; and so, strategies such as judicious use of immunosuppression with reduction when possible, cancer screening (dermatologic visits, regular pap smears) and avoidance of excessive sun exposure may promote early detection^[50,51]^.

## THE IMPACT OF CHECKPOINT IMMUNOTHERAPY FOLLOWING LIVER TRANSPLANTATION

The treatment of recurrent HCC in the LT recipient is a complex endeavor. Given the rates of multiorgan involvement with tumor recurrence in this population, there is a limit to the treatment modalities available^[13]^. Furthermore, after HCC recurrence, the overall survival at 5 years is about 50%, even with treatment^[52]^. Historically, treatment of HCC in the post-transplant patient has focused on the use of targeted therapies such as sorafenib with a demonstrated mortality benefit. Additionally, stereotactic body radiation therapy in localized bone disease and localized ablation and/or resection of solitary, small recurrent tumors are adjunctive treatments that can be employed^[14,53,54]^.

Consideration for the use of immunologic therapy in LT recipients is wrought with a complex interplay between the provision of adequate immunosuppression to protect the graft and augmentation of the immune response to detect and kill cancer cells. In addition to the typically reported immune-related adverse effects from ICI therapy such as hypophysitis, diarrhea/colitis and dermatitis, there is an additional risk of acute immune-mediated hepatitis in the liver allograft and an increased risk of acute cellular rejection (ACR)^[55]^.

Registry trials that led to the ultimate approval of ICI use for the treatment of HCC did not include liver (and other solid organ) transplant recipients as study participants. Hence, most of the data on the efficacy and safety of ICIs in these patients are drawn from case reports, case series and single center experiences^[56–60]^. A summary of some studies evaluating the efficacy and adverse events of these medications in the transplant population is summarized in Table 2. Relative safety, especially with close monitoring, has been described in a few case reports, but severe and sometimes fatal outcomes have also been published^[56,57,59]^. Some of the main adverse effects to be considered, especially in an LT recipient, are that of venous (sub-distribution HR up to 1.36 depending on the agent) and arterial thrombosis^[61,62]^. Poor wound healing is also a concern given the overlapping cellular and molecular processes between wound healing and cancer; but this increased risk has not been apparent in studies, and ICIs have been suggested to be safe in the peri-operative period^[63–65]^. The severity of checkpoint inhibitor-induced injury in the allograft can vary, and it is unclear if the altered immunologic milieu associated with solid organ transplant (SOT) and need for chronic immunosuppression play a role in the incidence and severity of this phenomenon. The most commonly reported liver injury is hepatocellular injury, and this pattern of injury is predominant in those who have undergone SOT^[66]^. Moreover, complications beyond hepatocellular injury have been exposed. Our group reported a case of severe cholestatic disease in the allograft after the treatment of recurrent HCC with nivolumab during the post-transplant period. This patient had no evidence of ACR on liver biopsy, but died from complications related to the confluent hepatic necrosis, consequent synthetic dysfunction and concurrent esophagitis and gastrointestinal hemorrhage^[58]^.

The gravest concern regarding the use of ICIs in the post-transplant setting is related to severe graft rejection or even allograft failure. Initial reports documented rates of rejection in transplant recipients treated with ICIs, anywhere from 36% in LT to 54% in kidney transplant recipients^[67]^. Systematic reviews have evaluated the risk of rejection in SOT recipients treated with ICIs^[60,68]^. These reviews are quite heterogenous: including a mix of SOT recipients with a variety of solid tumor malignancies. One single-center analysis of 17 SOT (including 8 LT) recipients treated with ICIs found that 18% of patients had acute allograft rejection, a cumulative incidence of cancer progression of 50% at 6 months, and 65% mortality over the median follow up period of 4.6 months^[60]^. Another pooled analysis of 64 SOT recipients documented the rate of allograft rejection at 41% following checkpoint immunotherapy for malignancies in the post-transplant period. Of note, the highest risk of ACR was seen in those treated with PD-1 inhibitors, and the lowest risk was in those on CTLA-4 inhibitor therapy^[68]^. This finding supports the previously proposed theory that the PD-1 pathway could play a critical role in determining graft tolerance^[69]^. In an LT recipient cohort, Munker *et al*.^[70]^ carried out a systemic review of 14 cases of LT recipients treated with ICIs, with ACR reported in 29% of patients - and lethal outcomes in 75% of those with ACR.

It is important to note that ICIs have been investigated for use in the pre-transplant setting, with mixed outcomes. One study reported the use of a pre-transplant toripalimab (Anti-PD-1) with resultant post-transplant fatal acute hepatic necrosis^[71]^. Another case series of nivolumab use for pre-transplant tumor treatment reported the absence of allograft loss, tumor recurrence and death^[72]^. Though this is worth mentioning, the safety and efficacy of ICIs in the pre-transplant setting are quite broad and beyond the scope of this review^[73]^.

A variety of factors may affect the development of rejection of liver allograft during ICI therapy. The timing of ICI use has been implicated by a few studies. In the LT population, the use of ICIs in those with a median interval of ~2 to 8 years post-LT has been associated with little to no reports of ACR, but rejection seems much higher when used in the early post-transplant period, up to a year following transplantation^[74–76]^. This phenomenon may be explained by the development of transplant immunological tolerance, which refers to the decreased immune activity against the allograft, and thus reduced immunosuppression needs^[77]^. Being an immunologically privileged organ, liver allograft immune tolerance can occur many years post-LT and is evidenced by maturation and depletion of self-reactive T-cells, progressive upregulation of CD4+ regulatory T-cells (which can suppress the injurious activity of Th cells) and regulatory dendritic cells, as well as the ongoing interaction of these alloreactive cells with hepatocytes and cholangiocytes in the allograft^[78,79]^. This reduced immune activity against the allograft is responsible for the decline in basal immune activity in those with more remote transplantation as opposed to those in the early post-transplant period, hence the importance of considering the time interval from LT in the use of ICIs.

Modification of immunosuppression in LT recipients undergoing treatment with ICI is somewhat unclear. Data suggests that immunosuppression could be reduced in LT patients prior to starting ICIs; as a robust T-cell response is required for successful activity of ICIs and less stringent immunosuppression could facilitate this^[28,80]^. Different approaches to achieving this goal have been documented. The use of prophylactic corticosteroids in a patient maintained on Tacrolimus and Everolimus, with close monitoring was reported in a case by Biondani *et al*.^[56]^. This patient was managed successfully with no evidence of ACR or IRAEs. Systemic corticosteroids serve as a potent treatment for IRAEs or ACR, and this may explain the utility of their use^[67]^. De Toni *et al*.^[57]^ reported a patient who was managed by progressive tapering of immunosuppression while being closely monitored on ICIs with no apparent adverse effects. A few studies showed improved survival in patients managed with ICIs and mTOR inhibitors. Compared to the CNIs, the mTOR inhibitors have been postulated as having significant anti-neoplastic, anti-angiogenetic and anti-proliferative properties, related to the selective inhibition of protein synthesis required for cancer cell growth and proliferation, with the induction of G1 cell cycle arrest, promoting cancer cell apoptosis, decreased translation of DNA damage, as well as restoration of radiosensitivity in some radioresistant tumors^[81,82]^. It has been suggested that these properties may be responsible for the improved survival shown in these studies^[83,84]^. However, all these observations are based on case reports and small series, and there is no consensus recommendation for an immunosuppression strategy prior to initiating ICIs.

Interestingly, in a study reviewing liver biopsies of persons who were post-LT and on ICIs, Munker *et al*.^[70]^ demonstrated that liver biopsies with ACR had increased levels of PD-1 expression, whereas those without ACR did not have increased PD-1 expression. This suggested a relationship between PD-1 expression and risk of acute cellular rejection following treatment with ICIs that could be further studied to better implement treatment in these patients (as this study evaluated only seven samples)^[70]^.

## IMPORTANT CONSIDERATIONS FOR THE USE OF CHECKPOINT INHIBITORS AFTER LIVER TRANSPLANTATION

Although there are no societal recommendations on the strategies for the use of ICIs in the treatment of HCC in LT recipients, some guidance can be drawn from published studies. The timing of ICI use should be considered. Based on the limited data available, initiating ICIs should be approached with caution in the early years post-LT for HCC or other tumors^[74,75,85]^. However, the choice of agent or agent combinations should be guided primarily by the tumor type, as well as available data behind its safety, efficacy and response. Moreover, in the case of HCC, choosing ICIs should be considered after other systemic therapies have failed. Where possible, a liver biopsy should be performed prior to initiation of ICIs in post-transplant patients. Appropriate staining for, and measurement of, PD-1 expression can be performed. In this regard, overexpression of PD-1 may suggest an increased risk of rejection with PD-1 inhibitor use, and may therefore prompt consideration of anti-CTLA-4 therapy^[70]^. Limited data suggests that combination therapy with PD1/CTLA-4 inhibition can have lower rejection than monotherapy^[86]^. Moreover, anti-PD-1/PD-L1 monotherapy has been suggested to confer a higher risk of rejection than anti CTLA-4 monotherapy. This finding was suggested by an analysis of 12 post-LT recipients on ICI therapy with ACR occurring in 50% of the subjects on anti-PD1 therapy compared to none of those on anti-CTLA-4 therapy^[87]^. Also, a review of 34 published reports of ICI therapy in SOT recipients showed that 85% of the documented cases of ACR occurred in those on anti-PD1 therapy^[88]^. The putative explanation for this is the role of the PD1 pathway in the development of transplant immune tolerance based on its ability to alter the balance between pathogenic and regulatory T-cells^[89]^. It is important to note that another study of 28 LT recipients reported similar rates of ACR in both groups of subjects^[90]^. Nonetheless, most reports of post-transplant ICI use in the treatment of HCC are based on cases where anti PD-1 therapy was used (as it is preferred for HCC), making it difficult to assess if anti-PD-1 therapy (compared to anti-CTLA-4) has a higher risk of rejection, or if the findings are biased towards its higher use^[67,86]^. When starting therapy, the highest risk for graft rejection has been reported over the first 3 weeks of therapy, and close follow up should be implemented in this period^[86]^. The choice of immunosuppression in the LT recipient and the need for changes before commencing ICI are still debatable. At this time, there is no clear data to provide recommendations on if, and when to make changes, as data on corticosteroid pre-treatment or progressive tapering of immunosuppressive medications are limited^[56,57]^. Lastly, patient preferences should be considered during the selection of these therapeutic agents. Prior to initiating therapy with ICIs, all patients should be properly counseled and provided informed consent on the efficacy and risks of immune-related adverse effects, as well as the risk of acute cellular rejection and even potential graft failure. These discussions should ideally be carried out by both the treating oncologist as well as the transplant expert providing liver-related care.

## FUTURE DIRECTIONS

Treatment of HCC with ICI in LT recipients is an area of oncology, hepatology and transplant medicine that is actively advancing. Immunotherapy continues to develop beyond the use of checkpoint inhibitors to include adoptive cell therapy - especially engineered T-cell receptor (TCR) and chimeric antigen receptor (CAR) T-cell therapy. CAR-T-cells have been extensively used in hematological malignancies mainly due to the lack of antigen heterogeneity in heme-derived cells. However, CAR-T therapy is being evaluated in solid tumors, and CAR-T cells directed towards glypican-3 could possibly eliminate glypican-3 positive HCC cells - this is a promising future intervention^[91,92]^. Another encouraging modality of adoptive cell therapy involves the use of HBV-specific TCR therapy. In a study by Tan *et al*.^[93]^, it was shown that by utilizing the integrated short segments of HBV DNA in HCC cells, T-cells can be specifically engineered to recognize specific HBV epitopes, thus personalizing therapy. Following administration to two patients with metastatic HCC, one of the patients showed decreased size of most of his pulmonary metastasis^[93]^. The use of HBV-specific TCR therapy has therefore shown promise in the management of HBV-related HCC recurrence in the LT recipient^[94,95]^. The results of these adoptive cell therapies, though promising, need further research given the fine balance between optimal efficacy via robust T-cell activity and immunosuppression after LT^[94]^. The use of other potential approaches includes therapeutic vaccines against HCC tumor-associated antigens (such as glypican-3, alpha-feto protein), as well as the use of oncolytic viruses such as the orthoreovirus to modulate innate immune response^[96–98]^. More research into these novel methods is needed to determine the efficacy and safety of the LT recipient.

With the use of checkpoint inhibitors in the post-transplant population occurring more frequently, more randomized controlled trials evaluating its efficacy and safety in this specific population are necessary and essential. In this incredible era of precision medicine, further studies should lead to an understanding of ICIs in those on immunosuppressive medications, thus providing a framework for individually optimized therapy in the treatment of HCC in this population.

## Figures and Tables

**Table 1.** Immune checkpoint inhibitors in the systemic therapy of hepatocellular carcinoma

Trial	Therapy class	Study therapy	Comparison	Population	Endpoint
KEYNOTE-240^[30]^	PD1	Pembrolizumab	Placebo	Second line systemic therapy	OS, PFS
CheckMate 459^[31]^	PD1	Nivolumab	Sorafenib	First systemic therapy	OS, ORR, PFS
IMbrave 150^[32]^	PDL1/Anti-VEGF	Atezolizumab + Bevacizumab	Sorafenib	First systemic therapy	OS, PFS
ORIENT-32^[33]^ (NCT03794440)	PDL1/Anti-VEGF	Sintilimab + IBI305	Sorafenib	First systemic therapy	OS, ORR
CHECKMATE-9DW (NCT04039607)	PD1/CTLA4	Nivolumab + Ipilimumab	Sorafenib or Lenvatinib	First systemic therapy	OS
COSMIC-312 (NCT03755791)	PDL1/TKI	Atezolizumab + Cabozantinib	Cabozantinib *vs*. Sorafenib	First systemic therapy	PFS, OS
LEAP-002 (NCT03713593)	PD1/TKI	Pembrolizumab + Lenvatinib	Lenvatinib + Placebo	First systemic therapy	PFS, OS
RATIONALE-301 (NCT03412773)	PD1	Tislelizumab	Sorafenib	First systemic therapy	OS
HIMALAYA (NCT03298451)	PDL1/CTLA4	Durvalumab + Tremelimumab	Durvalumab *vs*. Sorafenib	First systemic therapy	OS
PHOCUS (NCT02562755)	VACCINE/TKI	Pexa-Vec (modified vaccine virus) + Sorafenib	Sorafenib	First systemic therapy	OS
KEYNOTE-937 (NCT03867084)	PD1	Pembrolizumab	Placebo	Radiological response following ablation or resection	OS, RFS
CHECKMATE-9DX (NCT03383458)	PD1	Nivolumab	Placebo	High recurrence risk following surgical resection or ablation	RFS
EMERALD-2 (NCT03847428)	PDL1/Anti-VEGF	Durvalumab + Bevacizumab	Durvalumab + placebo *vs*. Placebo + placebo	High recurrence risk following surgical resection or ablation	RFS
IMBRAVE-050 (NCT04102098)	PDL1/Anti-VEGF	Atezolizumab + Bevacizumab	Active surveillance	High recurrence risk following surgical resection or ablation	RFS
EMERALD-1 (NCT03778957)	PDL1/Anti-VEGF	TACE + Durvalumab + Bevacizumab	TACE + Durvalumab + placebo *vs*. TACE + placebo + placebo	First TACE	PFS
CHECKMATE-74W (NCT04340193)	PD1/CTLA4	TACE + Nivolumab + Ipilimumab	TACE + Nivolumab + placebo *vs*. TACE + placebo + placebo	First TACE	TTTP, OS
LEAP-012 (NCT04246177)	PD1/CTLA4	TACE + Pembrolizumab + Lenvatinib	TACE + placebo + placebo	First TACE	PFS, OS
TACE-3 (NCT04268888)	PD1	Drug-eluting bead TACE + Nivolumab	Drug-eluting bead TACE	First TACE	OS

**Table 2.** Efficacy and adverse events noted with immune checkpoint inhibitors in the liver transplant recipient

Study	Study type	Number of patients	Type of ICI	Sot type	Major adverse findings
Biondani *et al*.^[56]^	Case report (Letter to the editor)	1	Nivolumab	Liver transplant	Patient had no adverse effects; suggesting that pre-emptive corticosteroids and the combination of tacrolimus and everolimus may have prevented hepatic immune-related adverse events
De Toni *et al*.^[57]^	Case report (Letter to the editor)	1	Nivolumab	Liver transplant	No adverse effects were seen suggesting that treatment with checkpoint inhibitors under close surveillance of liver function might be feasible in select transplant recipients
Anugwom *et al*.^[58]^	Case report	1	Nivolumab	Liver transplant	Patient developed cholestatic disease in the allograft, with fatal confluent hepatic necrosis, consequent synthetic dysfunction, severe esophagitis and gastrointestinal hemorrhage
Owoyemi *et al*.^[60]^	Retrospective study	17	Nivolumab (53%), Pembrolizumab (24%), Cemiplimab (12%), Atezolizumab (6%)	Mixed SOT (7 KT, 8 LT, 2 OHT)	25% (2) of LT recipients suffered ACR 29% (2) of KT recipients had IRAEs (allograft rejection and colitis) One OHT recipient (50%) developed acute heart failure and died from presumed nivolumab cardiotoxicity
Abdel-Wahab *et al*.^[66]^	Retrospective study	39	Pembrolizumab (44%), Nivolumab (36%)*, Ipilimumab (36%)*	Mixed SOT 23 KT, 11 LT, 5 OHT	ACR seen in 49% KT recipients, 20% OHT recipients and 36% LT recipients Overall death in 46% of cases due to allograft rejection or rejection complication (4 KT, 3 LT, 1 OHT)
Gassmann *et al*.^[67]^	Case report	1	Nivolumab	Liver transplant	Severe cellular graft rejection, consequent decline in liver function and severe coagulopathy and fatal intracranial hemorrhage.
Kumar *et al*.^[68]^	Case series	2	Pembrolizumab	Kidney transplant	Both patients developed acute cellular rejection, but grafts were salvaged
Tsung *et al*.^[83]^	Retrospective study	7	Cemiplimab (86%), Pembrolizumab (14%)	Mixed SOT (4 KT, 2 LT, 1 lung transplant)	1 (100%) lung transplant recipient developed steroid-responsive pneumonitis 1 (25%) KT recipient developed progressive renal injury Preserved allograft function and no adverse effects were seen in those (3 patients) who received prophylactic steroids (all patients underwent minimization or conversion of CNI to mTOR inhibitors)

* Combination ipilimumab and nivolumab 3%. ICI: Immune checkpoint inhibitor; SOT: solid organ transplant; KT: kidney transplant; LT: liver transplant; OHT: orthotopic heart transplant; ACR: acute cellular rejection; IRAEs: immune-related adverse effects; CNI: calcineurin inhibitor; mTOR: mechanistic target of rapamycin.

## References

[1] Petrick JL, Florio AA, Znaor A, International trends in hepatocellular carcinoma incidence, 1978–2012. Int J Cancer 2020;147:317–30. 31597196 10.1002/ijc.32723 PMC7470451

[2] Bray F, Ferlay J, Soerjomataram I, Siegel RL, Torre LA, Jemal A. Global cancer statistics 2018: GLOBOCAN estimates of incidence and mortality worldwide for 36 cancers in 185 countries. CA Cancer J Clin 2018;68:394–424. 30207593 10.3322/caac.21492

[3] McGlynn KA, Petrick JL, El-Serag HB. Epidemiology of hepatocellular carcinoma. Hepatology 2021;73 Suppl 1:4–13. 10.1002/hep.31288 PMC7577946 32319693

[4] Mittal S, El-Serag HB. Epidemiology of hepatocellular carcinoma: consider the population. J Clin Gastroenterol 2013;47 Suppl:S2–6. 23632345 10.1097/MCG.0b013e3182872f29 PMC3683119

[5] Kew MC. Hepatocellular carcinoma: epidemiology and risk factors. J Hepatocell Carcinoma 2014;1:115–25. 27508181 10.2147/JHC.S44381 PMC4918271

[6] Mazzaferro V, Regalia E, Doci R, Liver transplantation for the treatment of small hepatocellular carcinomas in patients with cirrhosis. N Engl J Med 1996;334:693–9. 8594428 10.1056/NEJM199603143341104

[7] Marrero JA, Kulik LM, Sirlin CB, Diagnosis, staging, and management of hepatocellular carcinoma: 2018 practice guidance by the american association for the study of liver diseases. Hepatology 2018;68:723–50. 29624699 10.1002/hep.29913

[8] Cheung TT, Poon RT, Yuen WK, Long-term survival analysis of pure laparoscopic versus open hepatectomy for hepatocellular carcinoma in patients with cirrhosis: a single-center experience. Ann Surg 2013;257:506–11. 23299521 10.1097/SLA.0b013e31827b947a

[9] Vitale A, Peck-Radosavljevic M, Giannini EG, Personalized treatment of patients with very early hepatocellular carcinoma. J Hepatol 2017;66:412–23. 27677712 10.1016/j.jhep.2016.09.012

[10] Roayaie S, Schwartz JD, Sung MW, Recurrence of hepatocellular carcinoma after liver transplant: patterns and prognosis. Liver Transpl 2004;10:534–40. 15048797 10.1002/lt.20128

[11] Halazun KJ, Najjar M, Abdelmessih RM, Recurrence after liver transplantation for hepatocellular carcinoma: a new MORAL to the story. Ann Surg 2017;265:557–64. 27611615 10.1097/SLA.0000000000001966

[12] de’Angelis N, Landi F, Carra MC, Azoulay D. Managements of recurrent hepatocellular carcinoma after liver transplantation: a systematic review. World J Gastroenterol 2015;21:11185–98. 26494973 10.3748/wjg.v21.i39.11185 PMC4607916

[13] Bodzin AS, Lunsford KE, Markovic D, Harlander-Locke MP, Busuttil RW, Agopian VG. Predicting mortality in patients developing recurrent hepatocellular carcinoma after liver transplantation: impact of treatment modality and recurrence characteristics. Ann Surg 2017;266:118–25. 27433914 10.1097/SLA.0000000000001894

[14] Guerrini GP, Berretta M, Tarantino G, Multimodal oncological approach in patients affected by recurrent hepatocellular carcinoma after liver transplantation. Eur Rev Med Pharmacol Sci 2017;21:3421–35. 28829499

[15] Couzin J. Cancer immunotherapy. Select T cells, given space, shrink tumors. Science 2002;297:1973. 10.1126/science.297.5589.1973a 12242411

[16] Mariotto AB, Yabroff KR, Shao Y, Feuer EJ, Brown ML. Projections of the cost of cancer care in the United States: 2010–2020. J Natl Cancer Inst 2011;103:117–28. 21228314 10.1093/jnci/djq495 PMC3107566

[17] Cheng A, Kang Y, Chen Z, Efficacy and safety of sorafenib in patients in the Asia-Pacific region with advanced hepatocellular carcinoma: a phase III randomised, double-blind, placebo-controlled trial. Lancet Oncol 2009;10:25–34. 19095497 10.1016/S1470-2045(08)70285-7

[18] Llovet JM, Ricci S, Mazzaferro V, ; SHARP Investigators Study Group. Sorafenib in advanced hepatocellular carcinoma. N Engl J Med 2008;359:378–90. 18650514 10.1056/NEJMoa0708857

[19] Lué A, Serrano MT, Bustamante FJ, Neutrophil-to-lymphocyte ratio predicts survival in European patients with hepatocellular carcinoma administered sorafenib. Oncotarget 2017;8:103077–86. 29262546 10.18632/oncotarget.21528 PMC5732712

[20] Sultan A, Anugwom CM, Wondifraw Z, Braimoh GA, Bane A, Debes JD. Single center analysis of therapy and outcomes of hepatocellular carcinoma in Sub-Saharan Africa. Expert Rev Gastroenterol Hepatol 2020;14:1007–11. 32730120 10.1080/17474124.2020.1802246 PMC7544626

[21] Wilhelm SM, Carter C, Tang L, BAY 43–9006 exhibits broad spectrum oral antitumor activity and targets the RAF/MEK/ERK pathway and receptor tyrosine kinases involved in tumor progression and angiogenesis. Cancer Res 2004;64:7099–109. 15466206 10.1158/0008-5472.CAN-04-1443

[22] Bruix J, Qin S, Merle P, Regorafenib for patients with hepatocellular carcinoma who progressed on sorafenib treatment (RESORCE): a randomised, double-blind, placebo-controlled, phase 3 trial. Lancet 2017;389:56–66. 27932229 10.1016/S0140-6736(16)32453-9

[23] Abou-Alfa GK, Meyer T, Cheng AL, Cabozantinib in patients with advanced and progressing hepatocellular carcinoma. N Engl J Med 2018;379:54–63. 29972759 10.1056/NEJMoa1717002 PMC7523244

[24] Kudo M, Finn RS, Qin S, Lenvatinib versus sorafenib in first-line treatment of patients with unresectable hepatocellular carcinoma: a randomised phase 3 non-inferiority trial. Lancet 2018;391:1163–73. 29433850 10.1016/S0140-6736(18)30207-1

[25] Zhu AX, Park JO, Ryoo B, Ramucirumab versus placebo as second-line treatment in patients with advanced hepatocellular carcinoma following first-line therapy with sorafenib (REACH): a randomised, double-blind, multicentre, phase 3 trial. Lancet Oncol 2015;16:859–70. 26095784 10.1016/S1470-2045(15)00050-9

[26] Yoong KF, McNab G, Hübscher SG, Adams DH. Vascular adhesion protein-1 and ICAM-1 support the adhesion of tumor-infiltrating lymphocytes to tumor endothelium in human hepatocellular carcinoma. J Immunol 1998;160:3978–88. 9558106

[27] Hui E. Immune checkpoint inhibitors. J Cell Biol 2019;218:740–1. 30760493 10.1083/jcb.201810035 PMC6400575

[28] Chen L, Flies DB. Molecular mechanisms of T cell co-stimulation and co-inhibition. Nat Rev Immunol 2013;13:227–42. 23470321 10.1038/nri3405 PMC3786574

[29] Gutierrez C, McEvoy C, Reynolds D, Nates JL. Toxicity of immunotherapeutic agents. Crit Care Clin 2021;37:605–24. 34053709 10.1016/j.ccc.2021.03.004

[30] El-khoueiry AB, Sangro B, Yau T, Nivolumab in patients with advanced hepatocellular carcinoma (CheckMate 040): an open-label, non-comparative, phase 1/2 dose escalation and expansion trial. Lancet 2017;389:2492–502. 28434648 10.1016/S0140-6736(17)31046-2 PMC7539326

[31] Finn RS, Ryoo BY, Merle P, ; KEYNOTE-240 investigators. Pembrolizumab as second-line therapy in patients with advanced hepatocellular carcinoma in KEYNOTE-240: a randomized, double-blind, phase III trial. J Clin Oncol 2020;38:193–202. 31790344 10.1200/JCO.19.01307

[32] Yau T, Park J, Finn R, CheckMate 459: A randomized, multi-center phase III study of nivolumab (NIVO) vs sorafenib (SOR) as first-line (1L) treatment in patients (pts) with advanced hepatocellular carcinoma (aHCC). Ann Oncol 2019;30:v874–5.

[33] Finn RS, Qin S, Ikeda M, ; IMbrave150 Investigators. Atezolizumab plus bevacizumab in unresectable hepatocellular carcinoma. N Engl J Med 2020;382:1894–905. 32402160 10.1056/NEJMoa1915745

[34] Ren Z, Fan J, Xu J, LBA2 Sintilimab plus bevacizumab biosimilar vs sorafenib as first-line treatment for advanced hepatocellular carcinoma (ORIENT-32)2. Ann Oncol 2020;31:S1287.

[35] Sangro B, Sarobe P, Hervás-Stubbs S, Melero I. Advances in immunotherapy for hepatocellular carcinoma. Nat Rev Gastroenterol Hepatol 2021;18:525–43. 33850328 10.1038/s41575-021-00438-0 PMC8042636

[36] Huang H, Lu Y, Zhou T, Gu G, Xia Q. Innate immune cells in immune tolerance after liver transplantation. Front Immunol 2018;9:2401. 30473690 10.3389/fimmu.2018.02401 PMC6237933

[37] Thomson AW, Knolle PA. Antigen-presenting cell function in the tolerogenic liver environment. Nat Rev Immunol 2010;10:753–66. 20972472 10.1038/nri2858

[38] de la Garza RG, Sarobe P, Merino J, Trial of complete weaning from immunosuppression for liver transplant recipients: factors predictive of tolerance. Liver Transpl 2013;19:937–44. 23784747 10.1002/lt.23686

[39] Feng S, Bucuvalas JC, Mazariegos GV, Efficacy and safety of immunosuppression withdrawal in pediatric liver transplant recipients: moving toward personalized management. Hepatology 2021;73:1985–2004. 32786149 10.1002/hep.31520 PMC12105584

[40] Starzl TE, Marchioro TL, Vonkaulla KN, Hermann G, Brittain RS, Waddell WR. Homotransplantation of the liver in humans. Surg Gynecol Obstet 1963;117:659–76. 14100514 PMC2634660

[41] Starzl TE, Marchioro TL, Waddell WR. The reversal of rejection in human renal homografts with subsequent development of homograft tolerance. Surg Gynecol Obstet 1963;117:385–95. 14065716 PMC2581919

[42] Eason JD, Loss GE, Blazek J, Nair S, Mason AL. Steroid-free liver transplantation using rabbit antithymocyte globulin induction: results of a prospective randomized trial. Liver Transpl 2001;7:693–7. 11510013 10.1053/jlts.2001.26353

[43] Anugwom CM, Parekh JR, Hwang C, MacConmara M, Lee WM, Leventhal TM. Comparison of clinical outcomes of induction regimens in patients undergoing liver transplantation for acute liver failure. Liver Transpl 2021;27:27–33. 32578297 10.1002/lt.25832

[44] Cangemi M, Montico B, Faè DA, Steffan A, Dolcetti R. Dissecting the multiplicity of immune effects of immunosuppressive drugs to better predict the risk of de novo malignancies in solid organ transplant patients. Front Oncol 2019;9:160. 30972289 10.3389/fonc.2019.00160 PMC6445870

[45] Wai LE, Fujiki M, Takeda S, Martinez OM, Krams SM. Rapamycin, but not cyclosporine or FK506, alters natural killer cell function. Transplantation 2008;85:145–9. 18192925 10.1097/01.tp.0000296817.28053.7b PMC4084728

[46] Colic M, Stojic-Vukanic Z, Pavlovic B, Jandric D, Stefanoska I. Mycophenolate mofetil inhibits differentiation, maturation and allostimulatory function of human monocyte-derived dendritic cells. Clin Exp Immunol 2003;134:63–9. 12974756 10.1046/j.1365-2249.2003.02269.x PMC1808848

[47] Bhat M, Mara K, Dierkhising R, Watt KD. Gender, race and disease etiology predict de novo malignancy risk after liver transplantation: insights for future individualized cancer screening guidance. Transplantation 2019;103:91–100. 29377876 10.1097/TP.0000000000002113

[48] Mathur A, Franco ES, Leone JP, Obesity portends increased morbidity and earlier recurrence following liver transplantation for hepatocellular carcinoma. HPB (Oxford) 2013;15:504–10. 23750492 10.1111/j.1477-2574.2012.00602.x PMC3692019

[49] Liang W, Wang D, Ling X, Sirolimus-based immunosuppression in liver transplantation for hepatocellular carcinoma: a meta-analysis. Liver Transpl 2012;18:62–9. 21964956 10.1002/lt.22441

[50] Jiang Y, Villeneuve PJ, Fenton SS, Schaubel DE, Lilly L, Mao Y. Liver transplantation and subsequent risk of cancer: findings from a Canadian cohort study. Liver Transpl 2008;14:1588–97. 18975293 10.1002/lt.21554

[51] Haagsma EB, Hagens VE, Schaapveld M, Increased cancer risk after liver transplantation: a population-based study. J Hepatol 2001;34:84–91. 11211912 10.1016/s0168-8278(00)00077-5

[52] Sapisochin G, Goldaracena N, Astete S, Benefit of treating hepatocellular carcinoma recurrence after liver transplantation and analysis of prognostic factors for survival in a large Euro-American series. Ann Surg Oncol 2015;22:2286–94. 25472651 10.1245/s10434-014-4273-6

[53] Schlitt HJ, Neipp M, Weimann A, Recurrence patterns of hepatocellular and fibrolamellar carcinoma after liver transplantation. J Clin Oncol 1999;17:324–31. 10458250 10.1200/JCO.1999.17.1.324

[54] Gomez-Martin C, Bustamante J, Castroagudin JF, Efficacy and safety of sorafenib in combination with mammalian target of rapamycin inhibitors for recurrent hepatocellular carcinoma after liver transplantation. Liver Transpl 2012;18:45–52. 21932373 10.1002/lt.22434

[55] Haanen JBAG, Carbonnel F, Robert C, ; ESMO Guidelines Committee. Management of toxicities from immunotherapy: ESMO Clinical Practice Guidelines for diagnosis, treatment and follow-up. Ann Oncol 2017;28:iv119–42. 28881921 10.1093/annonc/mdx225

[56] Biondani P, De Martin E, Samuel D. Safety of an anti-PD-1 immune checkpoint inhibitor in a liver transplant recipient. Ann Oncol 2018;29:286–7. 29293878 10.1093/annonc/mdx548

[57] De Toni EN, Gerbes AL. Tapering of immunosuppression and sustained treatment with nivolumab in a liver transplant recipient. Gastroenterology 2017;152:1631–3. 28384452 10.1053/j.gastro.2017.01.063

[58] Anugwom C, Leventhal T. Nivolumab-induced autoimmune-like cholestatic hepatitis in a liver transplant recipient. ACG Case Rep J 2020;7:e00416. 32766358 10.14309/crj.0000000000000416 PMC7363460

[59] Friend BD, Venick RS, McDiarmid SV, Fatal orthotopic liver transplant organ rejection induced by a checkpoint inhibitor in two patients with refractory, metastatic hepatocellular carcinoma. Pediatr Blood Cancer 2017;64:e26682. 10.1002/pbc.26682 28643391

[60] Owoyemi I, Vaughan LE, Costello CM, Clinical outcomes of solid organ transplant recipients with metastatic cancers who are treated with immune checkpoint inhibitors: a single-center analysis. Cancer 2020;126:4780–7. 32786022 10.1002/cncr.33134 PMC8772343

[61] Wang TF, Khorana AA, Carrier M. Thrombotic complications associated with immune checkpoint inhibitors. Cancers (Basel) 2021;13:4606. 34572833 10.3390/cancers13184606 PMC8469452

[62] Moik F, Chan WE, Wiedemann S, Incidence, risk factors, and outcomes of venous and arterial thromboembolism in immune checkpoint inhibitor therapy. Blood 2021;137:1669–78. 33067632 10.1182/blood.2020007878 PMC8016631

[63] López-Cortés A, Abarca E, Silva L, Identification of key proteins in the signaling crossroads between wound healing and cancer hallmark phenotypes. Sci Rep 2021;11:17245. 34446793 10.1038/s41598-021-96750-5 PMC8390472

[64] Elias AW, Kasi PM, Stauffer JA, The feasibility and safety of surgery in patients receiving immune checkpoint inhibitors: a retrospective study. Front Oncol 2017;7:121. 28660171 10.3389/fonc.2017.00121 PMC5466999

[65] Sun J, Kirichenko DA, Chung JL, Perioperative outcomes of melanoma patients undergoing surgery after receiving immunotherapy or targeted therapy. World J Surg 2020;44:1283–93. 31811340 10.1007/s00268-019-05314-2

[66] Abdel-Wahab N, Safa H, Abudayyeh A, Checkpoint inhibitor therapy for cancer in solid organ transplantation recipients: an institutional experience and a systematic review of the literature. J Immunother Cancer 2019;7:106. 30992053 10.1186/s40425-019-0585-1 PMC6469201

[67] Gassmann D, Weiler S, Mertens JC, Liver allograft failure after nivolumab treatment-a case report with systematic literature research. Transplant Direct 2018;4:e376. 30255136 10.1097/TXD.0000000000000814 PMC6092180

[68] Kumar V, Shinagare AB, Rennke HG, The safety and efficacy of checkpoint inhibitors in transplant recipients: a case series and systematic review of literature. Oncologist 2020;25:505–14. 32043699 10.1634/theoncologist.2019-0659 PMC7288631

[69] Blazar BR, Carreno BM, Panoskaltsis-Mortari A, Blockade of programmed death-1 engagement accelerates graft-versus-host disease lethality by an IFN-gamma-dependent mechanism. J Immunol 2003;171:1272–7. 12874215 10.4049/jimmunol.171.3.1272

[70] Munker S, De Toni EN. Use of checkpoint inhibitors in liver transplant recipients. United European Gastroenterol J 2018;6:970–3. 10.1177/2050640618774631 PMC6137599 30228883

[71] Chen GH, Wang GB, Huang F, Pretransplant use of toripalimab for hepatocellular carcinoma resulting in fatal acute hepatic necrosis in the immediate postoperative period. Transpl Immunol 2021;66:101386. 33744409 10.1016/j.trim.2021.101386

[72] Tabrizian P, Florman SS, Schwartz ME. PD-1 inhibitor as bridge therapy to liver transplantation? Am J Transplant 2021;21:1979–80. 33316117 10.1111/ajt.16448

[73] Qiao ZY, Zhang ZJ, Lv ZC, Neoadjuvant programmed cell death 1 (PD-1) inhibitor treatment in patients with hepatocellular carcinoma before liver transplant: a cohort study and literature review. Front Immunol 2021;12:653437. 34349755 10.3389/fimmu.2021.653437 PMC8326904

[74] DeLeon TT, Salomao MA, Aqel BA, Pilot evaluation of PD-1 inhibition in metastatic cancer patients with a history of liver transplantation: the Mayo Clinic experience. J Gastrointest Oncol 2018;9:1054–62. 30603124 10.21037/jgo.2018.07.05 PMC6286929

[75] Dueland S, Guren TK, Boberg KM, Acute liver graft rejection after ipilimumab therapy. Ann Oncol 2017;28:2619–20. 28961840 10.1093/annonc/mdx281

[76] Herbaux C, Gauthier J, Brice P, Efficacy and tolerability of nivolumab after allogeneic transplantation for relapsed Hodgkin lymphoma. Blood 2017;129:2471–8. 28270452 10.1182/blood-2016-11-749556

[77] Lechler RI, Sykes M, Thomson AW, Turka LA. Organ transplantation--how much of the promise has been realized? Nat Med 2005;11:605–13. 15937473 10.1038/nm1251

[78] Lei H, Reinke P, Volk HD, Lv Y, Wu R. Mechanisms of immune tolerance in liver transplantation-crosstalk between alloreactive T cells and liver cells with therapeutic prospects. Front Immunol 2019;10:2667. 31803188 10.3389/fimmu.2019.02667 PMC6877506

[79] Du X, Chang S, Guo W, Zhang S, Chen ZK. Progress in liver transplant tolerance and tolerance-inducing cellular therapies. Front Immunol 2020;11:1326. 32670292 10.3389/fimmu.2020.01326 PMC7326808

[80] Varkaris A, Lewis DW, Nugent FW. Preserved liver transplant after PD-1 pathway inhibitor for hepatocellular carcinoma. Am J Gastroenterol 2017;112:1895–6. 29215617 10.1038/ajg.2017.387

[81] Hua H, Kong Q, Zhang H, Wang J, Luo T, Jiang Y. Targeting mTOR for cancer therapy. J Hematol Oncol 2019;12:71. 31277692 10.1186/s13045-019-0754-1 PMC6612215

[82] Zheng Y, Jiang Y. mTOR inhibitors at a glance. Mol Cell Pharmacol 2015;7:15–20. 27134695 PMC4849280

[83] Tsung I, Worden FP, Fontana RJ. A pilot study of checkpoint inhibitors in solid organ transplant recipients with metastatic cutaneous squamous cell carcinoma. Oncologist 2021;26:133–8. 32969143 10.1002/onco.13539 PMC7873324

[84] Rao RD, Buckner JC, Sarkaria JN. Mammalian target of rapamycin (mTOR) inhibitors as anti-cancer agents. Curr Cancer Drug Targets 2004;4:621–35. 15578919 10.2174/1568009043332718

[85] Smedman TM, Line PD, Guren TK, Dueland S.Graft rejection after immune checkpoint inhibitor therapy in solid organ transplant recipients. Acta Oncol 2018;57:1414–8. 29912605 10.1080/0284186X.2018.1479069

[86] Nguyen LS, Ortuno S, Lebrun-Vignes B, Transplant rejections associated with immune checkpoint inhibitors: a pharmacovigilance study and systematic literature review. Eur J Cancer 2021;148:36–47. 33721705 10.1016/j.ejca.2021.01.038

[87] Kittai AS, Oldham H, Cetnar J, Taylor M. Immune checkpoint inhibitors in organ transplant patients. J Immunother 2017;40:277–81. 28719552 10.1097/CJI.0000000000000180

[88] Ros J, Matos I, Martin-Liberal J. Immunotherapy in organ-transplanted cancer patients: efficacy and risk of organ rejection. Ann Oncol 2019;30:1173–7. 30977776 10.1093/annonc/mdz129

[89] Tanaka K, Albin MJ, Yuan X, PDL1 is required for peripheral transplantation tolerance and protection from chronic allograft rejection. J Immunol 2007;179:5204–10. 17911605 10.4049/jimmunol.179.8.5204 PMC2291549

[90] Au KP, Chok KSH. Immunotherapy after liver transplantation: where are we now? World J Gastrointest Surg 2021;13:1267–78. 34754394 10.4240/wjgs.v13.i10.1267 PMC8554723

[91] Gao H, Li K, Tu H, Development of T cells redirected to glypican-3 for the treatment of hepatocellular carcinoma. Clin Cancer Res 2014;20:6418–28. 25320357 10.1158/1078-0432.CCR-14-1170

[92] Jiang Z, Jiang X, Chen S, Anti-GPC3-CAR T cells suppress the growth of tumor cells in patient-derived xenografts of hepatocellular carcinoma. Front Immunol 2016;7:690. 28123387 10.3389/fimmu.2016.00690 PMC5225101

[93] Tan AT, Yang N, Lee Krishnamoorthy T, Use of expression profiles of HBV-DNA integrated into genomes of hepatocellular carcinoma cells to select T cells for immunotherapy. Gastroenterology 2019;156:1862–76.e9. 30711630 10.1053/j.gastro.2019.01.251

[94] Hafezi M, Tan A, Bertoletti A. Personalized armored TCR-redirected T cell therapy for liver/organ transplant with recurrent cancer. Cells 2021;10:1861. 34440630 10.3390/cells10081861 PMC8393584

[95] Qasim W, Brunetto M, Gehring AJ, Immunotherapy of HCC metastases with autologous T cell receptor redirected T cells, targeting HBsAg in a liver transplant patient. J Hepatol 2015;62:486–91. 25308176 10.1016/j.jhep.2014.10.001

[96] Tagliamonte M, Petrizzo A, Mauriello A, Tornesello ML, Buonaguro FM, Buonaguro L. Potentiating cancer vaccine efficacy in liver cancer. Oncoimmunology 2018;7:e1488564. 30288355 10.1080/2162402X.2018.1488564 PMC6169594

[97] Mizukoshi E, Nakamoto Y, Tsuji H, Yamashita T, Kaneko S. Identification of alpha-fetoprotein-derived peptides recognized by cytotoxic T lymphocytes in HLA-A24+ patients with hepatocellular carcinoma. Int J Cancer 2006;118:1194–204. 16152611 10.1002/ijc.21468

[98] Samson A, Bentham MJ, Scott K, Oncolytic reovirus as a combined antiviral and anti-tumour agent for the treatment of liver cancer. Gut 2018;67:562–73. 27902444 10.1136/gutjnl-2016-312009 PMC5868283

